# Case of juvenile dermatomyositis (JDM), thrombotic thrombocytopenic purpura (TTP), and Purtscher retinopathy

**DOI:** 10.1186/1546-0096-10-S1-A66

**Published:** 2012-07-13

**Authors:** Ofra Goldzweig, Kabita Nanda, Shanail Berry, Hulya Bukulmez

**Affiliations:** 1 MetroHealth Medical Center, Pepper Pike, OH, USA; 2 MetroHealth Medical Center, Cleveland, OH, USA; 3 Rainbow Babies and Children, Cleveland, OH, USA

## Purpose

The aim of this report is to present a patient with juvenile dermatomyositis (JDM) who developed TTP and retinopathy and showed a dramatic response to B cell ablating therapy with rituximab. Concurrence of JDM, TTP, and retinopathy in a child has never been reported.

## Methods

Here we describe a previously healthy and athletic 12 year old female who was diagnosed with JDM following a period of two weeks of pruritic rash, fever, headaches, muscle pain and weakness. Her work-up was significant for elevated muscle enzymes and an abnormal MRI of bilateral quadriceps that demonstrated increased signal intensity. The patient was hospitalized for intravenous pulse methylprednisolone and intravenous immunoglobulin (IVIG) treatment. She showed significant improvement in her skin rash and muscle weakness, and was discharged with oral prednisone, naproxen and methotrexate. The patient returned to the hospital one week after discharge with complaints of blurry vision and headaches. In the emergency department the patient was noted to have retinal infarcts and was admitted directly to the Pediatric Intensive Care Unit (PICU). She was found to have thrombocytopenia, hemolytic anemia and renal failure. During her PICU stay she developed seizures and pulmonary edema and was subsequently diagnosed with TTP and severe retinopathy. Her ADAMTS-13 activity was found to be low (64%). She was treated with pulse steroid therapy, hemodialysis, plasmapheresis, multiple infusions of Fresh Frozen Plasma (FFP) and IVIG, but her condition continued to deteriorate. After four days of plasmapheresis and three days of hemodialysis she was given rituximab. Within 48 hours after rituximab treatment her condition dramatically improved. Rituximab was given at the same dose once weekly for 4 total doses, and no additional courses of rituximab were necessary.

## Results

The patient’s retinopathy showed improvement 21 days after first rituximab treatment and continued to improve gradually over the following year. Currently, the patient is in remission with maintenance therapy of cyclosporine, methotrexate, and IVIG for the past three years. She has no residual renal deficits, but continues to have ocular nerve atrophy with improvement of her retinal lesions.

## Conclusion

In conclusion, we presented a very rare case of a patient with JDM and TTP with Purtschners retinopathy. It is important to note that in retinopathy secondary to a vasculitic autoimmune disease with or without acquired TTP, the addition of IVIG to other immunosuppressants could be a key therapeutic tool. This case is important to discuss since it showed us the novel success of rituximab in halting JDM and TTP, and the effective treatment of Purtscher retinopathy with IVIG.

## Disclosure

Ofra Goldzweig: None; Kabita Nanda: None; Shanail Berry: None; Hulya Bukulmez: None.

